# Irrigation Scheduling for Maize under Different Hydrological Years in Heilongjiang Province, China

**DOI:** 10.3390/plants12081676

**Published:** 2023-04-17

**Authors:** Tangzhe Nie, Zhenping Gong, Zhongxue Zhang, Tianyi Wang, Nan Sun, Yi Tang, Peng Chen, Tiecheng Li, Shuai Yin, Mengmeng Zhang, Siwen Jiang

**Affiliations:** 1 Key Laboratory of Effective Utilization of Agricultural Water Resources, Ministry of Agriculture and Rural Affairs, Northeast Agricultural University, Harbin 150030, China; 2019036@hlju.edu.cn (T.N.);; 2 School of Water Conservancy and Electric Power, Heilongjiang University, Harbin 150006, China; 3 Post-doctoral Scientific Research Station of Crop Science, College of Agriculture, Northeast Agricultural University, Harbin 150030, China; 4 School of Water Conservancy and Civil Engineering, Northeast Agricultural University, Harbin 150030, China; 5 College of Agricultural Science and Engineering, Hohai University, Nanjing 210024, China; 6 Earth System Division, National Institute for Environmental Studies, Tsukuba 3058506, Japan; 7 Heilongjiang Provincial Key Laboratory of Ecological Restoration and Resource Utilization for Cold Region, School of Life Sciences, Heilongjiang University, Harbin 150006, China

**Keywords:** irrigation schedule, maize, crop water requirement, irrigation water requirement, hydrological year

## Abstract

Appropriate irrigation schedules could minimize the existing imbalance between agricultural water supply and crop water requirements (*ET*_c_), which is severely impacted by climate change. In this study, different hydrological years (a wet year, normal year, dry year, and an extremely dry year) in Heilongjiang Province were calculated by hydrological frequency methods. Then, the single crop coefficient method was used to calculate the maize *ET*_c_, based on the daily meteorological data of 26 meteorological stations in Heilongjiang Province from 1960 to 2020. Afterward, the CROPWAT model was used to calculate the effective precipitation (*P*_e_) and irrigation water requirement (*Ir*), and formulate the irrigation schedules of maize in Heilongjiang Province under different hydrological years. The results showed that *ET*_c_ and *Ir* decreased first and then increased from west to east. The *P*_e_ and crop water surplus deficit index increased first and then decreased from west to east in Heilongjiang Province. Meanwhile, the average values of the *Ir* in were 171.14 mm, 232.79 mm, 279.08 mm, and 334.47 mm in the wet year, normal year, dry year, and extremely dry year, respectively. Heilongjiang Province was divided into four irrigation zones according to the *Ir* of different hydrological years. Last, the irrigation quotas for the wet year, normal year, dry year, and extremely dry year were 0~180 mm, 20~240 mm, 60~300 mm, and 80~430 mm, respectively. This study provides reliable support for maize irrigation practices in Heilongjiang Province, China.

## 1. Introduction

Water scarcity has become a rising threat to the sustainability of agricultural systems, as water is one of the essential elements in agricultural production [1,2]. Seventy percent of the water utilized globally is allocated to agriculture, mostly for irrigation [3]. Due to inadequate field management and irrigation systems, a large proportion of water resources are wasted during irrigation [4]. Water shortages are becoming more of a problem in many regions of the world, as a result of climate change and the impact of population expansion on water demand for agricultural irrigation [5]. Water shortages are expected to be more serious under future climate change conditions [6]. Hence, meeting the irrigation water needs of crops has an impact on global food security, economic growth, and social stability. The appropriate management of irrigation water resources is essential to maintaining sustainable agricultural productivity [7].

Climate change and the expanding demands of the water-using industry are driving up the need for irrigation. Since the late 1990s, the vapor pressure deficit has been dramatically rising, along with a steady rise in temperature, which might trigger a rise in global evapotranspiration (*ET*) [8]. Climate change has also caused an increase in extreme precipitation, resulting in enhanced seasonal variability in runoff, making precipitation periods concentrated and dry periods longer [9]. Events such as extreme precipitation and increased *ET* have induced agricultural drought by depleting soil water supplies. Crop yields are greatly affected by agricultural droughts that occur throughout the growing season. [10]. When irrigation is needed, *ET* and precipitation offer a measurement of the amount of water used by the crop throughout the growing season, directing decisions on the rate of irrigation replenishment [11]. Prior research has shown that the irrigation water requirement (*Ir*) variation was significantly correlated with the *ET* and also affected by meteorological factors such as precipitation [12]. Irrigation has been noted to be able to moderate excessive precipitation and irregular precipitation variations during the crop-growing season by Pendergrass et al. [13]. Arid and semi-arid regions could benefit from an increase in the economic advantages of irrigation, which will also spread to wet and semi-humid regions to boost crop yields. [14]. The future climate predicted by Bisselink et al. for agricultural areas with a Mediterranean climate showed a trend toward less precipitation (less water available) and a trend toward more water requirements for crops, indicating that irrigation water supplies would become unreliable and require artificial recharge [15]. The analysis of regional crop water requirement patterns and the development of appropriate irrigation schedules is crucial to maintaining stable regional agricultural production [16].

The current imbalance between the agricultural water supply and requirements can be optimized by proper irrigation schedules [17]. Previous studies used hyperspectral reflectance data, crop evapotranspiration (*ET*_0_), and environmental variables for irrigation scheduling [18,19,20,21]. For example, Moseki et al. studied the crop water requirements (*ET*_c_) and *Ir* of *Jatropha curcas* in Botswana, responding to the uneven spatial and temporal distribution of precipitation occurring in the north and south of the country [22]. The most widely used approach for the simulation of irrigation schedules at the crop-field scale relies on the equations of the FAO-56 method [23]. In this case, the irrigation schedules simulated for different hydrological years were similar to most irrigation schedules in agricultural production. Variations in precipitation, air temperature, solar radiation, and precipitation distribution in different hydrologic years resulted in large inter- and intra-annual differences in the *Ir*, irrigation frequency, and irrigation timing [24]. Potopová et al. predicted the *Ir* for crops in the Czech Republic and calculated periods when extreme water use occurred; to relieve water use pressure, supplemental irrigation was needed [25]. José et al. analyzed the annual water allocation decisions under different hydrological drought scenarios for drought-prone southern Spain [26]. Alizadeh et al. studied the AquaCrop model’s efficiency for wheat in Karaj for 7- and 14-day irrigation periods, indicating that the model had a good ability in predicting grain yield [27]. In semi-arid regions of China, irrigation was able to relieve the effects of uneven precipitation [28]. In dry years, irrigation was more effective for crop growth than in wet years [29]. Ren et al. explored the optimal maize planting density for different hydrological years [30]. Nakayama studied the variation of *ET*_c_ and *Ir* of major crops under different precipitation in the Yellow River basin and analyzed the effect of irrigation on crops and the water cycle [31]. The study of irrigation schedules during the growth period of a given crop helped to improve the accuracy of regional agricultural water planning and relieve agricultural water pressure.

Identifying the changing patterns of regional *ET*_c_ is particularly important for irrigation system development, field water balance studies, and irrigation water management. Over the last 30 years, several models (SiSPAT, SVAT, CERRES) have been developed to simulate*ET*_c_*;* however, these models required input parameters that were not easily obtained at both the spatial and temporal scales [32,33]. In many studies, the *ET*_c_ was calculated by the Penman–Monteith method, with the single crop coefficient method recommended by the FAO-56 [34]. The FAO-56-recommended Penman–Monteith method is one of the most reliable methods used in the world and has been widely used to derive *ET*_c_*,* this method was favored due to its simplicity and robustness in practical applications [35]. The Penman–Monteith method requires less input data to obtain acceptable *ET*_c_ values compared to other models that require a large input of physical models [36], ground measurements [37], and satellite measurements [38]. Using the Penman–Monteith method would reduce the uncertainty caused when a meteorological factor is inaccurate or missing [39]. The study of the spatial and temporal distribution of *ET*_0_ in the region of Moldova showed a decreasing trend by the Penman–Monteith method. Arshad used the Penman–Monteith method to calculate the *ET*_c_ for Romania and the Mediterranean region for the period 1961–2099; the results showed that under the future increase in temperature and wind speed and decrease in precipitation and relative humidity, maize *ET*_c_ show an increasing trend [40]. Moreover, crop growth periods also have a close relationship with *ET*_c_ estimation [41].

Heilongjiang Province is one of the important grain regions in China, with total production amounting to 11.3% of China’s grain output [6]. Maize, as one of the major crops in Heilongjiang Province, has a planting area accounting for 44.83% of the grain planting area and 52.75% of the total grain production in Heilongjiang Province [41,42]. However, precipitation in the Heilongjiang region tends to increase in spring and decrease in summer and during the growth period of crops [6]. Heilongjiang Province has been under the influence of East Asian monsoon weather and experienced severe extreme drought and flood conditions since the 1970s. The spatial and temporal differences in climate and precipitation resulted in spatial and temporal differences in the *Ir* for maize in Heilongjiang Province [43]. Several researchers have studied the water supply and requirements of maize as well as its yield under climate change in Heilongjiang Province. Wang et al. found that the *Ir* illustrated a decreasing trend from the west to the east in Heilongjiang Province [44]. Nie et al. found that actual maize evapotranspiration showed an increasing trend from the north to the south, while the water use efficiency of maize showed a downward trend from the east to the west in Heilongjiang Province [41]. Based on the current water supply and requirement for maize in Heilongjiang Province, establishing the appropriate irrigation schedules for maize by zoning in the actual production and irrigation guidance to balance the spatial and temporal distribution of water resources will help guarantee food security in Heilongjiang Province.

The main objectives of this study were to (1) clarify the distribution pattern of *ET*_c_, effective precipitation (*Pe*), the water surplus and deficit index (*CWSDI*), and the *Ir* during the maize growth period in Heilongjiang Province in wet years, normal water years, dry years, and extremely dry years; and (2) formulate the irrigation schedules for maize in Heilongjiang Province in different hydrological years. The study results provide support for optimizing agricultural water resource allocation and ensuring food security in Heilongjiang Province.

## 2. Materials and Methods

### 2.1. The Study Area

**Figure 1:**
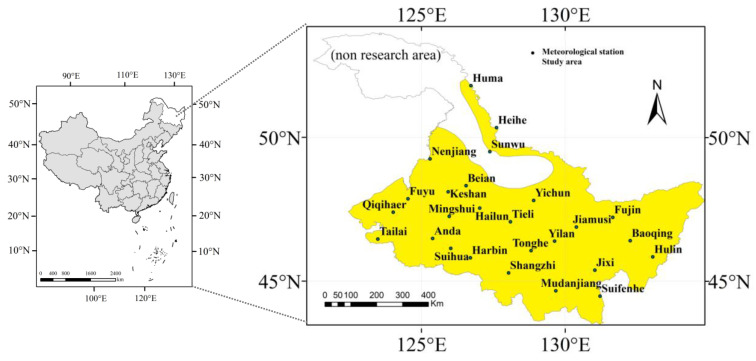
Locations of the study area and 26 meteorological stations.

This study used the daily meteorological data from 26 meteorological stations in Heilongjiang Province from 1960 to 2020, including the daily average relative humidity, daily average wind speed, daily sunshine hours, daily maximum air temperature, daily minimum air temperature, daily precipitation, and the longitude and latitude information of each meteorological station. The quality of the above data was carefully assessed, and the missing data were estimated using methods suggested by the FAO-56. All the above data were obtained from the China Meteorological Data Network (http://data.cma.cn, accessed on 30 March 2022). The meteorological data and the missing data were estimated by the method in FAO-56 [45]. The soil data in this study were from the China Soil Database (http://vdb3.soil.csdb.cn/, accessed on 30 March 2022). According to the Heilongjiang Provincial Agricultural Commission’s reports, “Heilongjiang Crop Variation Accumulative Temperature Zone” [46] and “Area Layout Planning of High-quality and High-yield Main Food Crops in Heilongjiang Province” in 2020 [47], the sixth accumulative temperature zone is not suitable for maize planting. Therefore, the sixth temperature accumulation zone was not studied in this study. Figure 1 shows the study area and the distribution of meteorological stations.

### 2.2. The Division of Hydrological Years

In order to estimate the rainfall deficit for irrigation water requirements, a statistical analysis was made from the long-term rainfall records. The definitions as the rainfall with a 90%, 75%, 50%, and 25% probability of exceedance represented the extremely dry years, dry years, normal years, and wet years, respectively. The four values were useful for the programming of the irrigation supply and the simulation of irrigation management conditions. 

For the programming of the irrigation water supply and management, the rainfall data of extremely dry years, dry years, normal years, and wet years were used. An estimate of the respective rainfall data was obtained by computing and plotting the probabilities from the rainfall records. The different steps involved were:

i. Tabulate the yearly rainfall totals for a given period.
ii. Arrange data in a descending order of magnitude.
iii. Tabulate the plotting position according to:

(1) $$Fa=\frac{100m}{n+1}$$

where *Fa* is the empirical frequency of m items in the observation series, *m* is the sequence number of the observation series arranged from large to small, and *n* is the number of years in the observation series.

iv. Plot values on a log-normal scale and obtain the logarithmic regression equation.
v. Calculate the year values at 90%, 75%, 50%, and 25% probability.
vi. Determine the monthly values for the dry year according to the following relationship:

(2) $$P_{idry}=P_{iav}\times \frac{P_{dry}}{P_{av}}$$

where *P_i_*_av_ is the average monthly rainfall for month *i*, *P_i_*_dry_ is the monthly rainfall dry year for month *i*, *P*_av_ is the average yearly rainfall, and *P_dry_* is the yearly rainfall at 75% probability of exceedance.

Similarly, the values for extremely dry years, normal, and wet years could also be determined by the method mentioned above.

### 2.3. CROPWAT 8.0 Model

CROPWAT 8.0 (FAO, Rome, Italy) is a crop decision support model developed by the Food and Agriculture Organization of the United Nations. Crop water requirements were calculated by CROPWAT 8.0, which is widely used in Asia, Europe, and Africa; details about CROPWAT 8.0 are shown in references [48,49].

### 2.4. Effective Precipitation

In this study, we used the method recommended by the Soil Conservation Agency of the U.S. Department of Agriculture to calculate the effective precipitation [50], as follows:

(3) $$P_{e}=\left\{\begin{array}{c} P(4.17-0.2P)/4.17\\ 4.17+0.1P \end{array}\right.\begin{array}{c} \left(P\leq 8.3\;mm\right)\\ \left(P>8.3\;mm\right) \end{array}$$

where *P*_e_ is effective precipitation (mm) and *P* is precipitation (mm).

### 2.5. The Maize Water Requirement

The maize *ET*_c_ was calculated by the single crop coefficient method [49]. The total maize *ET*_c_ was accumulated from the daily water requirement during the growth period based on the crop coefficient in different growth periods, as in Equation (4). The crop evapotranspiration under standard conditions, denoted *ET_c_s*, is the evapotranspiration from disease-free, well-fertilized crops, grown in large fields, under the optimum soil water conditions, and achieving full production under the given climatic conditions [49].

(4) $${ET}_{c}=K_{c}\times {ET}_{0}$$

where *ET*_c_ is the crop water requirement (mm), *K*_c_ is the crop coefficient, and *ET*_0_ is the reference crop evapotranspiration (mm).

In this study, the maize growth period was divided into four stages according to the FAO-56 (Table 1): *L_ini_* is from the seeding stage to the seven-leaf stage, *L_dev_* is from the seven-leaf stage to the heading stage, *L_mid_* is from the heading stage to the milking stage, and *L_late_* is from the milking stage to the maturing stage [50]. The *K*_c_ values were indicated in the FAO-56 at different growth stages of maize under standard conditions. *K*_c-ini_, *K*_c-mid_, and *K*_c-end_ were applied to represent the values of single crop coefficient *K*_c_ in the initial-growth stage, mid-growth stage, and harvest stage, respectively. The *K*_c_ values during the develop-growth stage and late-growth stage were interpolated. The *K*_c-ini_, *K*_c-mid_, and *K*_c-end_ recommended values were 0.3, 1.20, and 0.35, respectively. The *K*_c-ini_, *K*_c-mid_, and *K*_c-end_ values were corrected automatically in our previous study [44,48].

*ET*_0_ was calculated by the Penman–Monteith method recommended by the FAO, as in Equation (5).

(5) $${ET}_{0}=\frac{0.408\Delta \left(R_{n}-G\right)+\gamma \frac{900}{T+273T}u_{2}(e_{s}-e_{a})}{\Delta +\gamma (1+0.34u_{2})}$$

where *R*_n_ is the net radiation at the crop surface (MJ·(m^2^·d)^−1^), *G* is soil heat flux (MJ·(m^2^·d)^−1^), *T* is the average air temperature (°C), *u*_2_ is the wind speed measured at 2 m height (m·s^−1^), *e*_s_ − *e*_a_ is the vapor pressure deficit (KPa), Δ is the slope of the vapor pressure curve (KPa·°C^−1^), *γ* is the psychrometric constant (KPa·°C^−1^), and 900 is a conversion factor.

### 2.6. Crop Water Surplus Deficit Index

To accurately reflect the water requirements and the water supply status of maize, we constructed the water surplus deficit index (*CWSDI*) to describe the water surplus and deficit during the maize growth stage [51], as in Equation (6):

(6) $$CWSDI=\frac{P_{e}-{ET}_{c}}{{ET}_{c}}$$

where *CWSDI* is the crop water surplus deficit index.

### 2.7. Irrigation Water Requirement and Irrigation Schedule

To avoid deep leakage loss, the net irrigation amount should be less than or equal to the water consumption in the root zone when calculating the irrigation water requirements. According to previous studies, the soil moisture content suitable for maize growth was 55~80% of the field capacity [52]. In this simulation, irrigation was carried out to 80% of the field capacity, when the soil moisture content dropped to 55% of the field capacity. The total irrigation amount was the sum of each irrigation quota. The irrigation water requirement was calculated by the daily soil water balance in the CROPWAT 8.0 model, as in Equation (7):

(7) $$I_{r,i}=D_{r,i-1}+{ET}_{c}-D_{r,i}-P_{i}+{RO}_{i}+{DP}_{i}$$

where *I_r,i_* is the irrigation water requirement on day *i* (mm), *D_r_*_,*i* − 1_ is the water consumption of the root zone on day *_i_*_− 1_ (mm), *ET_c_* is the water requirement (mm), *D_r_*_,*i*_ is the water consumption in the root zone on day *i* (mm), *P_i_* is the precipitation on day *i* (mm), *RO_i_* is the surface runoff loss on day *i* (mm), and *DP_i_* is the water loss from the deep leakage in the root zone on day *i* (mm).

### 2.8. ET_c_ Calibration and Verification 

The field *ET*_c_ data used in this study were from a 2-year irrigation schedule experiment in the National Irrigation Experimental Center (45°43′09″ N, 126°36′35″ E, and altitude 140 m), located in Harbin, Heilongjiang Province. We selected T1 in 2014 for the calibration and T2 in 2015 for the verification of *ET*_c_ in CROPWAT. The calibration data and the verification data were composed of *ET*_c_ at four growth stages (the Emergence stage, Jointing stage, Tasseling stage, and Filling stage) and the whole growth period of maize (Table 2).

The statistical parameters, including normalized RMSE (CV(RMSE)), determination coefficient (R^2^), Willmott’s agreement index (d), and model efficiency coefficient (EF), were determined for the performance of CROPWAT 8.0.

(8) $$R^{2}=1-\frac{\sum {\left(yi-ŷi\right)}^{2}}{\sum {\left(yi-ŷ\right)}^{2}}$$

(9) $$CV\left(RMSE\right)=\frac{1}{Ō}\sqrt{\frac{\sum {\left(Pi-Oi\right)}^{2}}{n}}$$

(10) $$d=1-\frac{\sum {\left(Pi-Oi\right)}^{2}}{\sum {\left(\left|Pi-Ō\right|+\left|Oi-Ō\right|\right)}^{2}}$$

(11) $$EF=\frac{\sum_{i=1}^{n}{\left(Oi-Ō\right)}^{2}-\sum_{i=1}^{n}{\left(Si-Oi\right)}^{2}}{\sum_{i=1}^{n}{\left(Oi-Ō\right)}^{2}}$$

where yi is the actual value, ŷi is the simulated value, and ŷ is the mean value. Ō is the mean observations, pi is the simulated value, and Oi is the observed value. *n* means the research count. A simulation can be considered perfect if the CV(RMSE) is smaller than 10%, good if between 10 and 20%. The d range is 0–1, with 0 indicating a bad fit and 1 indicating a good fit between the simulated and observed data. The EF value is smaller than 1, and a positive value indicates that the simulated value better describes the measured data trend than the mean observations.

### 2.9. Data Processing

The Jenks ranking method in the Arcmap10.2 (ESRI, Redlands, CA, USA) classification toolbox was used to divide Heilongjiang Province into four irrigation zones based on the size of the maize *Ir* in Heilongjiang Province to guide the irrigation practice in each zone.

The spatial distribution maps of *ET*_0_, *ET*_c_, *P*e, and *CWSDI* in different hydrological years were drawn by the Arcmap10.2 toolbox. The ordinary Kriging method and inverse distance weight method of the Arcmap10.2 toolbox was applied to interpolate the value of *ET*_0_, *ET*_c_, *P*_e_, and *CWSDI*.

## 3. Results

### 3.1. Calibration and Verification of ET_c_

**Figure 2:**
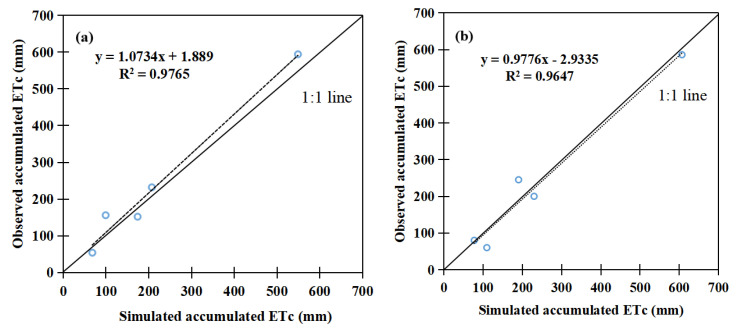
*ET*_c_ (**a**) calibration in 2014 and (**b**) validation in 2015 of CROPWAT model.

The model-simulated *ET*_c_ agreed well with the experimental observed data (Table 3). Each difference of the simulated and observed *ET*_c_ was less than 20 mm (Figure 2 and Table 3). Therefore, the CROPWAT model simulation results on maize *ET*_c_ during the whole growth stage were reliable and applicable for this study.

### 3.2. Maize Water Requirement (ET_c_) Spatial Distribution in Different Hydrological Years

**Figure 3:**
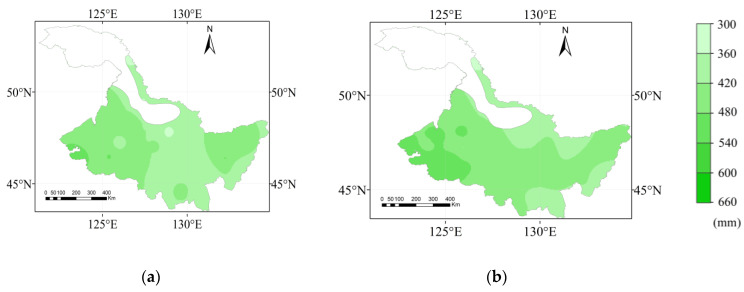

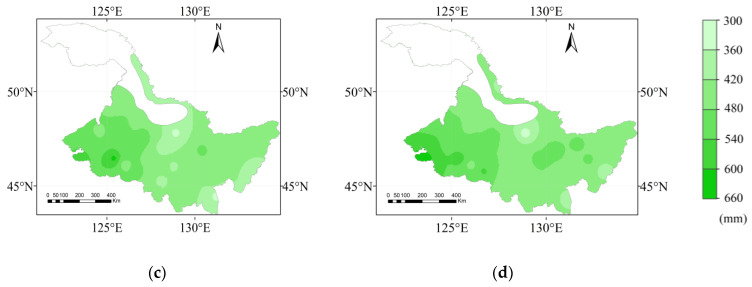
The spatial distribution of maize *ET*_c_ for a (**a**) wet year, (**b**) normal year, (**c**) dry year, and (**d**) extremely dry year.

The maize water requirements in Heilongjiang Province in different hydrological years are shown in Figure 3. The *ET*_c_ in the extremely dry, dry, normal, and wet years ranged from 651.00 to 340.60 mm, 610.40 to 349.40 mm, 593.00 to 366.80 mm, and 521.90 to 352.90 mm, respectively. The averages of the *ET*_c_ in extremely dry, dry, normal, and wet years were 478.64, 457.73, 440.71, and 420.74 mm, respectively. The high-value area of *ET*_c_ was located in the western region of Heilongjiang Province, and the change of *ET*_c_ in the high-value area was larger in 4 hydrological years. The change of the *ET*_c_ was more obvious in the western region in the extremely dry year. Tailai, the area with the largest *ET*_c_, increased by 129.1 mm in the extremely dry year compared with that in the wet year. The low-value area of the *ET*_c_ for maize was located in the middle part of the Heilongjiang Province, with rare change in 4 hydrological years.

### 3.3. Effective Precipitation (P_e_) Spatial Distribution in Different Hydrological Years

**Figure 4:**
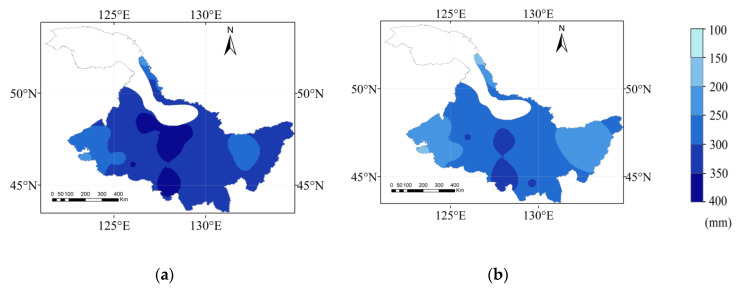

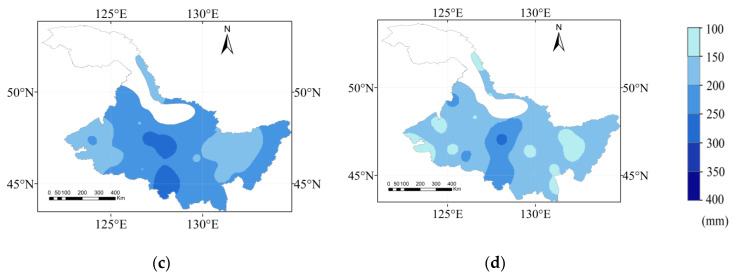
The spatial distribution of the *Pe* in the maize growth period for a (**a**) wet year, (**b**) normal year, (**c**) dry year, and (**d**) extremely dry year.

The *P*_e_ during the maize growth period in Heilongjiang Province in different hydrological years is shown in Figure 4. The *P*_e_ in the extremely dry, dry, normal, and wet years ranged from 126.90 to 277.30 mm, 152.60 to 294.90 mm, 185.20 to 339.70 mm, and 234.50 to 380.90 mm, respectively. The average of *P*_e_ in extremely dry, dry, normal, and wet years are 169.39, 212.20, 263.78, and 319.50 mm respectively. The *P*_e_ increased and then decreased from west to east during the maize growth period. The high-value area of *P*_e_ was mainly distributed in the central region, and the low-value area was located in the western and northern areas. The proportion of high *P*_e_ value area gradually decreases from wet years to extremely dry years. The four high-value areas of *P*_e_ in different years were mainly distributed in the south. The western region had the lowest *P*_e_ in the Heilongjiang Province in 4 hydrological years, and the area with the lowest *P*_e_ in the west in the extremely dry years was 150.70 mm.

### 3.4. The Maize Water Surplus and Deficit Index (CWSDI) Spatial Distribution in Different Hydrological Years

**Figure 5:**
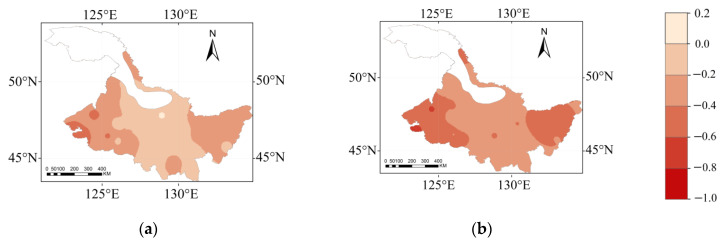

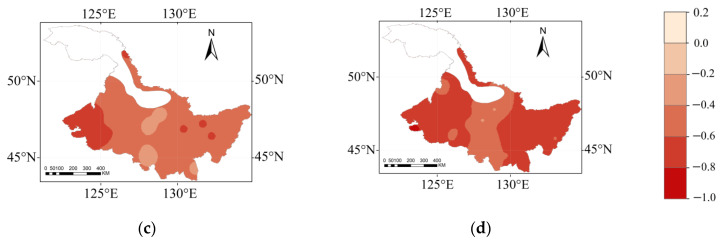
The spatial distribution of *CWSDI* in the maize growth period for a (**a**) wet year, (**b**) normal year, (**c**) dry year, and (**d**) extremely dry year.

The *CWSDI* of the maize growth period in Heilongjiang Province in different hydrological years is shown in Figure 5. The *CWSDI* in the extremely dry, dry, normal, and wet years ranged from −39.1 to −82.8%, −28.6 to −72.9%, −21.7 to −64.9%, and 18.8 to −55.1%, respectively. The *CWSDI* showed an increase and then a slight decrease from west to east in the wet years, as shown in Figure 5a. The water deficit area was mainly distributed in the western region, and the water surplus area was located in the northern region. From Figure 5b, the water deficit area in the normal year was mainly distributed in the west and a few eastern areas, and the water surplus area was located along the south. The *CWSDI* showed an increasing and then decreasing trend from west to east in the dry and extremely dry years, and the water deficit area was mainly located in the west and a few eastern areas, while the water surplus area was located in the south. From Figure 5d, the water deficit gradually developed from the east and west to the central region, and the water deficit area has gradually increased. The central region had a lower water deficit in the 4 hydrological years than the other regions. The western part of Heilongjiang Province might suffer from a serious water deficit during the 4 hydrological years without applying irrigation.

### 3.5. The Irrigation Water Requirement (Ir) Spatial Distribution in Different Hydrological Years

**Figure 6:**
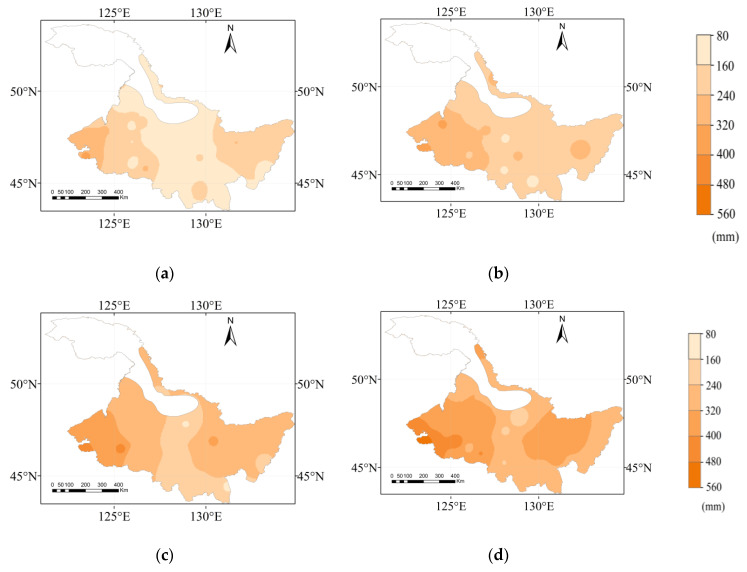
The spatial distribution of the *Ir* of maize for a (**a**) wet year, (**b**) normal year, (**c**) dry year, and (**d**) extremely dry year.

The *Ir* of maize in Heilongjiang Province in different hydrological years is shown in Figure 6. The *Irs* in the extremely dry, dry, normal, and wet years were from 82.20 to 332.10 mm, 144.20 to 340.60 mm, 136.70 mm to 437.60 mm, and 180.70 to 544.50 mm, respectively, with an average of 171.14, 232.79, 279.08, and 334.47 mm. The *Ir* decreased and then increased from west to east. The high-value area of *Ir* was mainly located in the western region, and the low-value area was located in the southern part of Heilongjiang Province. The high value of *ET*_c_ and low value of *P*_e_ were mainly distributed in the western region in different hydrological years; therefore, the *Ir* in the western region was in the high-value area in 4 hydrological years. The *Ir* low-value area was generally distributed in the central region.

### 3.6. The Irrigation Schedule in Different Hydrological Years

The study area was divided into four irrigation zones according to the different *Irs* of maize in Heilongjiang Province for the different hydrological years. In the wet years, the four irrigation zones were Zone I: 0~40 mm, Zone II: 40~90 mm, Zone III: 90~130 mm, and Zone IV: 130~180 mm. No irrigation was required in most areas of Zone I. The irrigation frequency in Zone II was 1~2 times, areas where two irrigation events were carried out, mainly in late May for the first irrigation and in mid-July to mid-August for the second irrigation. Zone II accounted for most of Heilongjiang Province. Zone III was mainly located in the western and a few eastern parts, while Zone IV was located in the western part.

**Figure 7:**
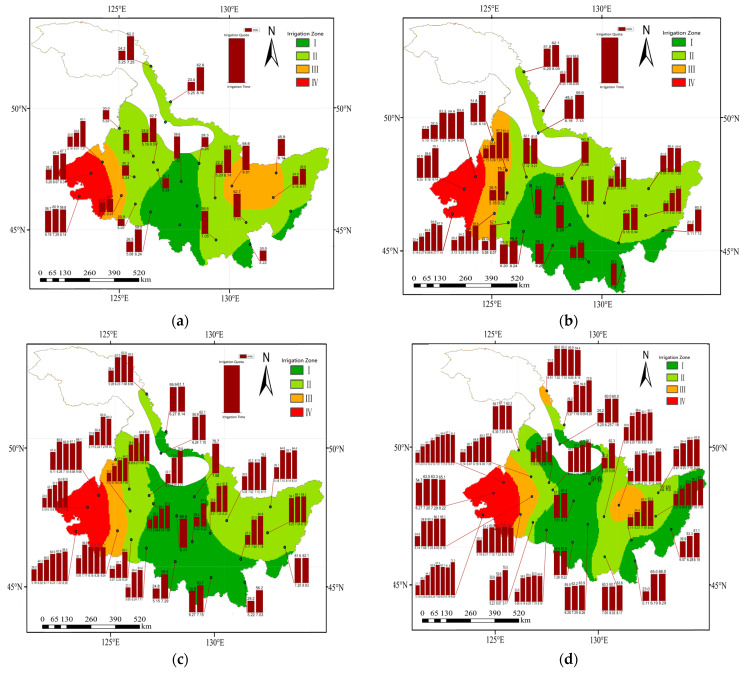
The spatial distribution of the irrigation schedules of maize for a (**a**) wet year, (**b**) normal year, (**c**) dry year, and (**d**) extremely dry year.

In the normal years, the study area was divided into Zone I: 0~110 mm, Zone II: 110~155 mm, Zone III: 155~200 mm, and Zone IV: 200~245 mm. Zone I was irrigated once or twice, with the irrigation mainly concentrated in June. Zone II was distributed in the eastern and central–western regions with two to four irrigation events. Zone III was distributed in the central–western region; it required corresponding irrigation in all of May (Figure 7b). Zone IV was located in the western region.

In the dry years, the irrigation zoning criteria for Heilongjiang Province were Zone I: 0~160 mm, Zone II: 160~215 mm, Zone III: 215~280 mm, and Zone IV: 280~340 mm. In the extremely dry years, the irrigation zoning criteria are were Zone I: 0~210 mm, Zone II: 210~270 mm, Zone III: 270~340 mm, and Zone IV: 340~450 mm. In the dry years and extremely dry years, Zone I was mainly distributed in the central region with 2~3 irrigation events, Zone II was distributed in the eastern and central–western regions, and Zone IV was located in the western region.

Compared to the wet years, the area of Zones I and IV expanded and the area of Zones II and III decreased in the normal water years. The number of irrigation events increased by 1–2 times in the other three Zones, except for Zone I, where there was no significant change, and most areas needed irrigation in July. Compared with the normal years, the area of Zone I increased significantly in the dry years, while the area of Zones III and IV did not change significantly. The frequency of the irrigation increased about 1~2 times, and the irrigation time in most areas was earlier than the normal years to the first time in May. The frequency of irrigation in dry years increased by about one to two times compared with normal years, and in most areas, the first irrigation was carried out earlier than in May. In the dry years, the first irrigation was mostly distributed in late May and early June. Compared with the dry years, the irrigation times increased in the extremely dry years, and the irrigation time was more intensive compared with the other three hydrological years.

### 3.7. Maize Irrigation Schedules at Typical Stations

Representative stations from the Qiqihar, Anda, Suihua, and Tonghe areas—distributed in Zones IV, III, II, and I, respectively—were chosen to analyze the variations in the irrigation schedules during the maize growth period in the study area (Table 4). The maize yield of the Qiqihar, Anda, Suihua, and Tonghe areas contributed 19.46%, 9.52%, 20.3%, and 10.03%, respectively, to the total maize production of the Heilongjiang Province in 2020. In Heilongjiang Province, the maize yield in the aforementioned four areas was crucial for food security.

In Qiqihar (in Zone IV), irrigation occurred three, five, six, and four times, with total irrigation amounts of 145~160 mm, 180~195 mm, 230~255 mm, and 230~250 mm in the wet, normal, dry, and extremely dry years, respectively. The frequency of irrigation in June increased in the dry years compared with the wet years, and there was no significant change in the single irrigation quota. More priority should be given to irrigation during the early and mid-growth periods due to the potential for spring droughts and low precipitation in Qiqihar.

Anda (in Zone III) received three, four, five, and five irrigation events totaling 60~70 mm, 150~170 mm, 260~285 mm, and 305–335 mm in the wet, normal, dry, and extremely dry years, respectively. Anda has low precipitation in spring [33], thus, even though maize needs less water during its early growth stage, it should concentrate on the appropriate spring irrigation to supplement soil moisture.

The total irrigation amounts in Suihua (in Zone II) were 20~25 mm, 80~90 mm, 110~125 mm, and 180~195 mm in the wet, normal, dry, and extremely dry years, respectively, with one, two, three, and three irrigation events. While the total irrigation amount for Tonghe (in Zone I) was 0 mm, 60–70 mm, 80–90 mm, and 175–190 mm in the normal, dry, and extremely dry years, respectively, with zero, one, two, and three irrigation events. Due to the low precipitation in the dry and extremely dry years in Tonghe, adequate irrigation should be carried out to ensure crop growth.

## 4. Discussion

The high-value areas of *Ir* in all 4 hydrological years in this study were mainly distributed in the western part of Heilongjiang Province [53]. The *P*_e_ was, in general, lower in the east than in the west; however, the *ET*_c_ was lower in the east during the maize growth period [6]. The low values of *ET*_c_ resulted in the maize *Ir* being generally higher in the eastern region than in central region and lower in the western region. In this study, *ET*_c_ high-value regions were all distributed in the western region for 4 different hydrological years. The *CWSDI* results for 4 different hydrological years showed that the maize in Heilongjiang Province was in a water deficit situation during the growth period in general, and some parts of the western and eastern regions were in a more severe water deficit situation. Previous studies on the water requirements of maize in Heilongjiang Province have shown that the *Ir* was higher in the western part of Heilongjiang Province [53]. Zhang et al. reported that the *ET*_c_ in the eastern maize growing area of Heilongjiang Province had grown higher, pointing out that the drought situation in the eastern region needs to be taken seriously [54]. Moreover, the *Ir* was higher in the central–eastern region (Jiamusi City) in the wet and extremely dry years compared with other surrounding areas. The irrigation division of the Jiamusi area was divided into Zone III, which might be caused by the lower *P*_e_ in the eastern part. In summary, the spatial distribution of the maize *Ir* in Heilongjiang Province was not uniform and appropriate irrigation schedules that could be applied during the maize growing period need to be filled [39]. The actual irrigation practice should take into account not only the *Ir* of maize, but also the water source and topographic conditions in the study area [55]. The irrigation schedules were implemented to provide a theoretical basis for the development of regional agricultural water use planning.

Liu et al. studied the spatial distribution of the *Ir* of major crops in China, suggesting that the average *ET*_c_ was 300~500 mm and *Ir* was 100~150 mm per year during the growing period of maize in Heilongjiang Province [56]. However, their study did not formulate irrigation schedules for the different hydrological years. The average *ET*_c_ in Heilongjiang province during 4 different hydrological years of maize growing periods in this study belonged to the same range. In this study, the *Ir* was higher than the results of Liu et al. This study and Liu et al. calculated the *Ir* and *P*_e_ using different calculation formulas. In this study, the *P*_e_ was calculated using the formula recommended by the USDA Soil Conservation Agency [48], and the *Ir* was calculated based on the process of soil root moisture dynamics. However, Liu et al. only used the difference between the *ET*_c_ and *P*_e_ to calculate the *Ir*. In addition, this study considered the initial soil water content in each region of Heilongjiang Province. The irrigation schedules for the different hydrological years were formulated, which was more guiding for the regional agricultural production. Li et al. studied the water consumption rules of maize in Harbin experimentally, indicating that the *Ir* of maize ranged from 200 to 380 mm [57]. In this study, the net irrigation water requirement of Harbin for the same hydrological year was 310 mm. According to the previous study, the irrigation efficiency was 70~80%; therefore, the *Ir* of Harbin was 388~420 mm. The simulated results of *Ir* in this study were slightly higher than the results of Li et al. due to the different irrigation limits chosen between the two studies. The *Ir* in this study was referred to as the net irrigation amount. In the western arid area, combined with the actual situation in Heilongjiang Province, permanent irrigation measures should be established to develop an efficient and water-saving agriculture. The diversion of water and the establishment of the corresponding irrigation district construction should be made in the area of the Nengjiang River and the Nilchi Reservoir, where surface water resources are relatively abundant. The well irrigation should be taken in other areas within this study according to the local water resource conditions to prevent overexploitation [58]. In agricultural production, farmers should make adjustments according to the actual irrigation water utilization coefficient of the local agricultural production.

Yang et al. calculated the extreme weather changes during the maize growth period in northeast China and pointed out that the frequency of droughts in the western and eastern parts of Heilongjiang Province was higher than that in the central part [59]. The results of this study showed that the degree of water deficit in different hydrological years in Heilongjiang Province was in the order of the western region > the eastern region > the central region. Maize in Heilongjiang Province was generally seeded in early May and harvested in late September, and the demand for soil moisture was more urgent during the growth period. A short period of drought will cause injury to maize growth and development [60]. In the early growth period, in particular, a short-term water deficit would cause serious yield reductions. Zhao et al. noted that the frequency of drought was highest in Heilongjiang Province during the early stages of maize growing periods [59]. Gao et al. also pointed out that crops in Heilongjiang Province were influenced by spring drought [51]. Zhang et al. reported that water requirements during the maize growing period in Zhaozhou, Heilongjiang Province, were influenced by the wind speed and sunshine hours, and supplemental irrigation of maize was required in the early growth stage to ensure proper germination and the emergence of maize [54]. The soil moisture during the period of maize planting has a great impact on maize growth [54]. Kang et al. pointed out that when irrigation water was insufficient, seed preservation, flower preservation, and moisture storage irrigation should be the main focus of irrigation [61]. In this study, the spring drought conditions were considered in the establishment of the irrigation schedule and the corresponding irrigation events were carried out in May in the areas where needed [60], as the CROPWAT 8.0 model has different settings of the planned irrigated soil layer in the different growth periods, resulting in less total available soil moisture at the initial growth stage of maize [49]. In this study, the irrigation schedules were calculated for the different hydrological years during the maize growth period in Heilongjiang Province. In agricultural production, farmers should also be guided to irrigate at the right time and amount, taking into account the actual local conditions including climate, soil, crop growth, and irrigation efficiency [62]. Water-saving technologies and soil moisture conservation measures for crops, such as plastic film mulching and straw incorporation, should be reasonably carried out [63]. Thus, the water use efficiency would be properly improved, while meeting the *ET*_c_ of maize. Additionally, the application of remote sensing technology in the observation of soil water content and crop evapotranspiration will be conducive to overcoming the spatial heterogeneity of irrigation [23].

## 5. Conclusions

The *ET*_c_ showed a general trend of decreasing and then increasing from west to east in four different hydrological years in Heilongjiang Province. The high-value areas of *P*_e_ were mainly located in the central part of Heilongjiang Province, where the precipitation was lower in the eastern and western parts in the dry and extremely dry years. *CWSDI* increased and then decreased from west to east, and the irrigation water shortage was serious in the eastern and western regions. The *Ir* showed a decreasing and then increasing trend from west to east during the maize growing period. The *Ir* and *CWSDI* indicated that in addition to the higher *ET*_c_ in the west, maize suffered water shortage more easily. The irrigation zone divisions in Heilongjiang Province are divided for different hydrological years according to the *Ir*, in which the total irrigation amount of each zone was in the order of Zone I < Zone II < Zone III < Zone IV. Zone I was mainly located in the central and northern regions and was mostly rain fed. Zone II was located mainly in the northeastern region and required attention in spring drought and timely supplementary irrigation. Zone III was mainly located in the western and some eastern areas, and Zone IV was mainly located in the western areas, which were the main irrigation areas and were irrigated several times depending on the different hydrological years. Moreover, an appropriate irrigation schedule in conjunction with reasonable agronomic practices and water-saving irrigation technologies will increase the effectiveness of irrigation water utilization.

## Figures and Tables

**Table 1:** Division of the maize growth period.

Maize Growth Period	Dividing Mode
Initial-growth stage (*L*_ini_)	Ground cover from seeding to 10% ground coverage
Develop-growth stage (*L*_dev_)	From 10% ground coverage to full coverage
Mid-growth stage (*L*_mid_)	From full coverage to maturity beginning
Late-growth stage (*L*_late_)	From maturity beginning to harvest or full senescence

**Table 2:** Irrigation schedule experiments in 2014 and 2015.

Year	Treatments	Irrigation Upper and Lower Limit in Different Growth Stages of Maize (% of FC)			
		Emergence Stage	Jointing Stage	Tasseling Stage	Filling Stage
2014	T1	80–100%	80–100%	80–100%	80–100%
2015	T2	100%	100%	100%	100%

Note: FC is the Field Capacity. The number before “–” in the table represents the lower limit of irrigation, and the number after “–” in the table represents the upper limit of irrigation. T1′s treatment of “80–100%” represents that the irrigation started when the soil moisture content reached 80% FC (the lower limit of irrigation), and the irrigation stopped when the soil moisture content reached 100% FC (the upper limit of irrigation). T2 represents the full irrigation treatments of maize growth.

**Table 3:** The fit indexes of the CROPWAT 8.0 model-simulated and measured *ET*_c_.

Parameters	CV (RMSE) (%)	d	R^2^	EF
*ET*_c_ in 2014	8.53	0.98	0.97	0.96
*ET*_c_ in 2015	9.86	0.98	0.96	0.96

**Table 4:** The maize irrigation schedules at Tonghe, Suihua, Anda, and Qiqihar.

Irrigation Zones	Stations	Hydrological Years	Irrigation Schedules			
			Total Irrigation Amount (mm)	Number of Irrigations	Irrigation Quota (mm)	Irrigation Date (Month.Day)
I	Tonghe	Wet year	0	0	0	-
		Normal year	60~70	1	60~70	6.20–7.10
		Dry year	80~90	2	25~30/55~60	5.20–5.31/6.20–6.30
		Extremely dry year	175~190	3	55~60/60~65/60~65	6.20–6.30/7.20–7.31/8.20–8.31
II	Suihua	Wet year	20~25	1	20~25	5.01–5.10
		Normal year	80~90	2	20~25/60~65	5.01–5.10/8.00–8.10
		Dry year	110~125	3	20~25/30~35/60~65	5.01–5.15/5.20–5.31/8.20–8.31
		Extremely dry year	180~195	3	50~55/55~60/75~80	6.20–6.30/8.00–8.10/9.10–9.20
III	Anda	Wet year	60~70	2	25~30/35~40	5.10–5.20/6.00–6.10
		Normal year	150~170	4	25~30/30~35/40~45/55~60	5.10–5.20/5.20–5.31/6.10–6.20/8.10–8.20
		Dry year	260~285	5	30~35/60~65/60~65/55~60/55~60	5.20–5.31/7.10–7.20/8.10–8.20/8.20–8.31/9.00–9.10
		Extremely dry year	305~335	6	25~30/55~60/55~60/55~60/60~65/55~60	5.10–5.20/6.15–6.25/7.05–7.15/7.20–7.31/8.10–8.20/8.31–9.10
IV	Qiqihar	Wet year	145~160	3	25~30/60~65/60~65	5.15–5.25/8.00–8.10/8.31–9.10
		Normal year	180~195	3	50~55/55~60/75~80	6.20–6.30/8.10–8.20/9.10–9.20
		Dry year	230~255	5	20~25/40~45/50~55/60~65/60~65	5.10–5.20/6.10–6.20/6.20–6.30/7.10–7.20/8.10–8.20
		Extremely dry year	230~250	4	50~55/60~65/60~65/60~65	6.20–6.31/7.05–7.15/7.20–7.31/8.20–8.30

## Data Availability

Not applicable.
